# Self-Supervised Object Distance Estimation Using a Monocular Camera

**DOI:** 10.3390/s22082936

**Published:** 2022-04-12

**Authors:** Hong Liang, Zizhen Ma, Qian Zhang

**Affiliations:** School of Computer Science and Technology, China University of Petroleum Huadong, Qingdao 266580, China; liangh@upc.edu.cn (H.L.); 20060075@upc.edu.cn (Q.Z.)

**Keywords:** object detection, monocular distance estimation, deep neural networks

## Abstract

Distance estimation using a monocular camera is one of the most classic tasks for computer vision. Current monocular distance estimating methods need a lot of data collection or they produce imprecise results. In this paper, we propose a network for both object detection and distance estimation. A network-based on ShuffleNet and YOLO is used to detect an object, and a self-supervised learning network is used to estimate distance. We calibrated the camera, and the calibrated parameters were integrated into the overall network. We also analyzed the parameter variation of the camera pose. Further, a multi-scale resolution is applied to improve estimation accuracy by enriching the expression ability of depth information. We validated the results of object detection and distance estimation on the KITTI dataset and demonstrated that our approach is efficient and accurate. Finally, we construct a dataset and conduct similar experiments to verify the generality of the network in other scenarios. The results show that our proposed methods outperform alternative approaches on object-specific distance estimation.

## 1. Introduction

With the development of deep learning in recent years, computer vision has taken a huge leap forward from traditional methods with this technology. Object detection [1], segmentation [2], and distance estimation are all tasks in this field. Although great efforts have been made to improve visual accuracy, the main focus has been on 2D vision tasks, such as object classification [3], detection [4], and segmentation [2]. In addition to achieve object detection, the distance between the camera and the recognized object (car, pedestrian, etc.) is now also critical. For 3D vision, such as real-time localization and map construction (SLAM) [5], augmented reality (AR) [6], robot navigation [7], artificial vision-based pose, and position estimation systems are very important. Distance estimation is one of these fundamental problems and has promising applications in many fields. In autonomous driving, this can provide important information to a car to prevent collisions. However, most of the current 3D vision work is usually solved by GPS, lidar [8], RGBD cameras, and binocular cameras [9,10]. Of the above methods, GPS, lidar, and RGBD are expensive, stereo systems usually require stereo calibration to obtain the relative position relationship between the two cameras, and the stereo calibration parameters cannot be changed throughout the process. The whole process is tedious and requires additional cameras.

**Figure 1:**
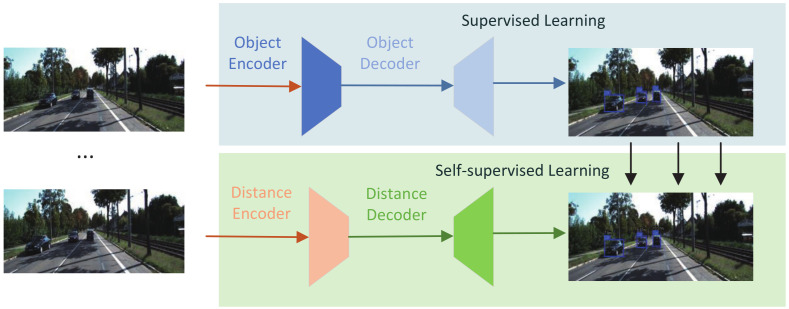
Overview of the joint prediction of distance and object detection from sequence images. Compared to previous approaches, our method can produce a more accurate result.

Therefore, an inexpensive solution is to use monocular cameras for object distance estimation. Monocular pose estimation was first pioneered by the work of Davidson et al. [11]. They recovered camera trajectories from monocular cameras by detecting natural landmarks using the Shi and Tomasi [12] operators. The method processes each image frame through the filter to jointly estimate map feature locations and camera poses. Monocular depth estimation was first used to determine the scene and geometric cues present in the image. Tuohy et al. used IPM to transform the image space into a bird’s eye view and then performed distance estimation [13]. However, the biggest problem with monocular depth estimation is how to recover depth as a scale factor. Ref. [14] used a camera as the primary sensor and an inertial system (imu) to determine the scale factor, and ref. [15] proposed to use geometric constraints between the road surface and the camera height to determine it. While the scale factor is a significant limitation because monocular vision loses the depth of field information, all feature points only have a 2D projection when they first appear. The actual position can appear at any line point between the optical center and the projection. The calculated distance usually has a significant error if we do not have the object’s actual size before the distance estimation. Only when the camera “moves” can we roughly estimate the distance. Therefore, continuous images are necessary for distance estimation. In recent years, it has become a trend to use a neural network to estimate the distance of monocular cameras. In previous work [16], depth was estimated using a convolutional neural network, and errors were reduced by training the network with successive images. Refs. [17,18,19] made network optimization for the depth estimation task. In this paper, we first use supervised training for object detection and then estimate object distances using self-supervised training. Before distance estimation, we need to know the size of the object. Therefore, this can only be used in highly controlled environments. For object detection, since we detect a few classes and have to perform subsequent distance estimation, in terms of saving resources, we propose Lite-Fast YOLOv5, which uses Shufflenetv2’s backbone network and significantly improves detection speed while maintaining slight loss inaccuracy. We combine camera movement and depth estimation for distance estimation, propose a new encoder–decoder network that introduces an attention mechanism into the decoder network, and use a multi-scale approach to generate depth images. We use a new reconstruction loss based on the previous L1 method in terms of the loss function. Figure 1 shows the methods we use in this paper.

The main contributions of this paper are the following:

1. We propose the Light-Fast YOLO network, combined with the multi-scale prediction of YOLOv5 and the light network Shufflenetv2, which reduces the number of parameters of the network and improves the speed without loss of much accuracy; we use the loss function combined with class, confidence and bounding box.
2. We propose a self-supervised method and new reconstruction loss function to resolve the unknown scale factor through sequence input images and use multi-scale-resolution depth maps with self-attention module to output detected objects’ distance.
3. We calibrate and analyze the camera and perform experiments on KITTI and CCP datasets to evaluate our method.

## 2. Related Work

In this section, we first review the study of traditional geometric methods of monocular object distance estimation. Afterwards, we discuss the methods combined with deep learning. At last, before introducing distance estimate, object detection is necessary.

### 2.1. Traditional Geometric Methods of Measuring Distance

In previous work, a traditional method for distance estimation is based on the image structure and camera parameters. Monocular cameras use focal length and matrix parameters for calculations, while binocular cameras use parallax calculations from two cameras at the same horizontal line. Liu et al. used two different focal lengths to estimate object distances, and this fusion ranging method is an excellent solution to the problem of inaccurate and challenging detection at long distances [20]. Tsai et al. obtained the image structure by detecting vanishing lines and extinction points in the image. However, the algorithm based on linear perspective is only suitable for scenes containing vanishing lines and extinction points, such as railways, roads, and streets [21]. Zhuo et al. used a method of obtaining image depth based on the degree of bokeh of a single image, which has the disadvantage that only the relative depth of the target in the image can be obtained, but not the absolute depth information of the target [22]. Ming et al. proposed a method based on occlusion cues to obtain relative depth information between targets by detecting occluded edges. However, this method is only applicable when there is an occlusion-obscured relationship between targets [23,24] that transforms the monocular problem into a binocular stereo matching problem. The above monocular ranging algorithms all require more or less specific depth cues, which are inefficient and error-prone with geometric methods.

### 2.2. Deep Learning Methods of Measuring Distance

Zhu et al. first proposed the use of labels with distance values in the training process, where the distance of a given object can be predicted directly on RGB images without the intervention of camera parameters [25]. Zhang et al. followed the [25] method to construct datasets and used both target detection and R-CNN-based deep regression networks for distance estimation. While these methods need to calculate the actual distance from velodyne point cloud, the cost is very expensive [26]. Xu et al. presented a U-net structured network for predicting dense depths through supervised learning, incorporating information from multi-scale layers and integration from a continuous conditional random field, rather than regressing directly on depth [27]. Ref. [28] considered the depth estimation model as a regression problem and trained the regression network by minimizing the mean square error. Kreuzig et al. used a recurrent convolutional neural network to determine the video sequence travel distance in an end-to-end manner [29]. Bian et al. acquired depth maps and ego-motion from image sequences in an unsupervised manner, which is identical to [30]. The deep-learning methods more or less do not use the camera matrix parameters. We propose a self-supervised learning-based approach for distance estimation and consider the camera parameters as part of the training.

### 2.3. Acquire Object

Before distance estimation, we first need to identify the object in the image. Target detection methods can be equally divided into traditional and neural network algorithms. Traditional object detection, such as [31,32,33], usually uses the method of manual feature extraction, including the following three steps: selection of a region of interest, feature extraction, and feature classification. However, using this method is computationally intensive, and the detection results are inaccurate. Convolutional-neural-network-based methods can be divided into anchor-based and anchor-free methods, with anchor-based learning based on real sticky note values. It is a qualitative leap from the previous method; two-stage includes [3,34,35], and one-stage includes [1,4,36,37]. Anchor-free does not use a bounding box when predicting from a boundary point or center point, including CornerNet [38] and CenterNet [39,40]. In the last two years, the same good results have been achieved with self-supervised target detection methods, which do not rely on real tagged value input, saving a lot of human and material resources, and learning to identify objects through machine self-supervision, such as [41,42] based on transformer [43]. model, based on pretrained target detection backbone for fine-tuning [44,45] which uses contrast learning. In this work, we first use LF-YOLO as our object detector, then we extract the object coordinates to the next step to predict the distance.

## 3. Materials and Methods

### 3.1. Light-Fast YOLO

The backbone network of YOLOv5s has many convolution layers, such as BottleneckCSP. One BottleneckCSP includes three convolution operations and a path with Bottleneck. The latter also includes some Conv layers, and this structure takes a long time in feature extraction. The speed of the network’s forward propagation is slow. While the Shufflenetv2’s [46] structure is similar to the BottleneckCSP, to improve efficiency, we use it as our backbone. It uses the depth-separable convolution (DWconv) [47], which introduces channel shuffle that allows information to flow between channels. This network structure can map more channel features with lower computational complexity and memory loss.

**Figure 2:**
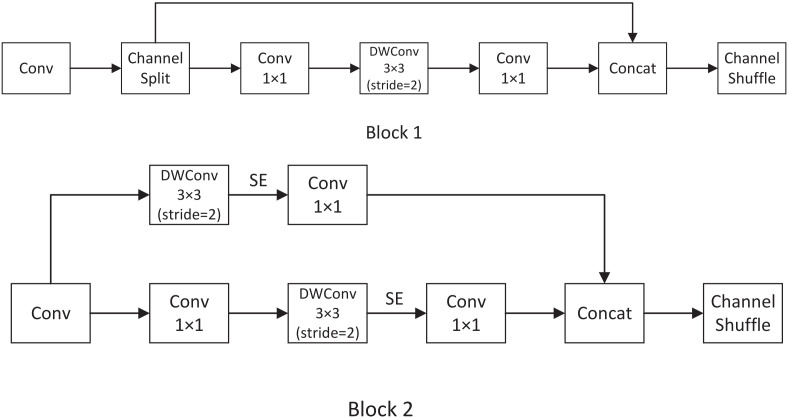
Shufflenetv2 module: we use different numbers of block 1 and block 2 in our network, and add SE layer in block 2.

As shown in Figure 2, the Shufflenetv2 unit is composed of two convolution blocks with DWconv. Shufflnetv2 introduces a new operation: channel split, block 1 shows that the channel dimension of feature map is split, the upper branch is mapped equally, the down branch includes three convolutions, and the input channels are equaled with the output channels, and the 1 × 1 block is not group convolution. The output of the two branches is no longer an add element, but concat together. This operation can ensure the flow of information. The modules in block 1 make it possible to channel split in the next unit. Block 2 is a downsample module without channel split. By copying input features and a convolution operation with a stride of 2, the feature size is reduced by half, and the number of channels is doubled when finally concat together. Based on block 2, we add an SE layer [48] after DWConv operation to better extract features. SE can make the model achieve better results by using the network to learn feature weights based on the loss so that effective feature maps are weighted heavily, and ineffective or less effective ones are weighted less.

**Figure 3:**
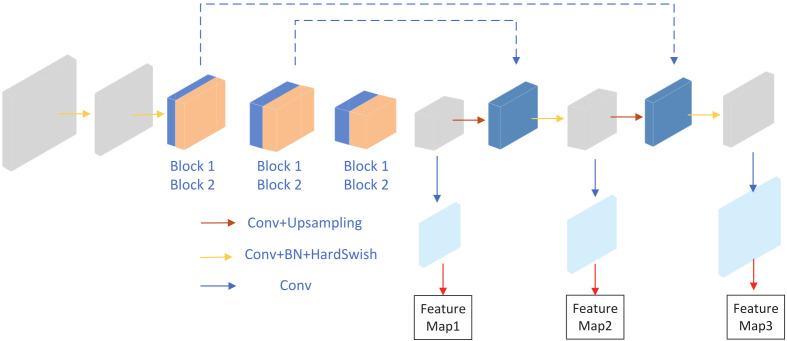
LF-YOLO. The backbone is based on Shufflenetv2, the head part use less BottleneckCSP module in YOLOv5.

Our proposed network is shown in Figure 3, it is based on YOLOv5s and combines the light network ShuffleNetv2. The backbone network uses the Shufflenetv2 structure. To avoid too many operations of slices, we remove the FOCUS module. In the head part, we reserve the multi-scale prediction method, the network outputs prediction tensors at three different scales, we cut part of the head module of YOLOv5s, and reduce the number of bottleneckCSP.

#### YOLO Loss Function

Object detection models such as YOLOv5 are accurate and can successfully identify objects in images as long as they are given enough samples. Our monocular visual ranging requires very accurate object bounding boxes. During training, the neural network uses loss functions and backpropagation to continuously update the model parameters and reduce model losses to improve detection accuracy. The loss function of LF-YOLOv5 is referenced from YOLOv5 and consists of three components: bounding box prediction $L_{bbox}$, confidence prediction $L_{obj}$, and classification prediction $L_{cls}$. Confidence and classification losses use BCElogits and BCEcls; for bounding boxes losses we choose EIoU [49] rather than CIoU used in YOLOv5. The difference in aspect ratio represented by v in CIoU is not the true difference between the width and height, respectively, and its confidence level, which sometimes prevents the model from optimizing similarity effectively. EIoU splits the aspect ratio and adds focal aggregation of good-quality anchor boxes, accelerating convergence and improving regression accuracy. This loss contains an overlap loss, a central distance loss, and a width and height loss. The first two parts continue the approach in CIoU, but the width and height loss directly minimizes the difference between the width and height of the target and anchor boxes, making convergence faster. *G* represents the prediction bounding box, $G_{t}$ represents the true bounding box, *c* represents a minimum rectangular area that can cover the two bounding boxes, $\rho_{G}^{2}$ represents the $G^{\prime }$ center, $G{}_{t}$’s Euclidean distance, and $c_{1}^{2}$, $\rho_{w}^{2}$, and $\rho_{h}^{2}$ represent the Euclidean distance between the height and width prediction box and the ground truth box, respectively. $c_{1}^{2}$ represents the diagonal length of *c*, while $c_{w}^{2}$ and $c_{h}^{2}$ represent height and width diagonal lengths of their minimum rectangular areas, respectively.

(1) $$L_{bbox}=EIoU=1-IoU+\frac{\rho_{G}^{2}}{c_{1}^{2}}+\frac{\rho_{w}^{2}}{c_{w}^{2}}+\frac{\rho_{h}^{2}}{c_{h}^{2}}$$

(2) $$\begin{array}{c} Loss=L_{cls}+L_{obj}+L_{bbox} \end{array}$$

### 3.2. Self-Supervised Scale-Aware Networks

**Figure 4:**
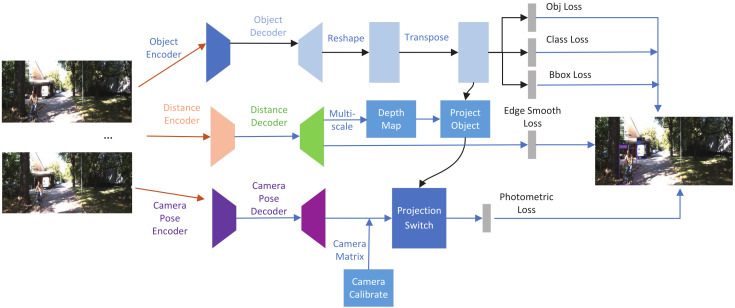
Overview of our proposed framework for the joint prediction of distance and object detection. The first row describes the prediction of the object detection, the second row describes the steps for the depth estimation, and the third row describes camera pose parameters.

In early self-supervised learning, Zhou et al.’s [30] first proposed structure-from-motion (SfM); it aimed at two tasks: (1) a monocular depth model predicting a scale-ambiguous depth $\hat{D}=g_{d}\left(I_{t}\left(p\right)\right)$ in the target image $I_{t}$, *p* represents per-pixel; (2) an ego-motion predictor $g_{x}:\left(I_{t},I_{t^{\prime }}\to I_{t\to t^{\prime }}\right)$ predicting a six-degrees-of-freedom rigid transformation. A limitation of this approach is that both depth and pose are estimated up to an unknown scale factor in the monocular SfM pipeline. Per-pixel may exist at a large number of possible incorrect depths; the traditional method is to use the viewpoints of $I_{t-1}$ and $I_{t+1}$ to estimate the appearance of a target image $I_{t}$ on camera images. Ref. [50] first conducted a study related to distance estimation on a fisheye camera using a similar method. We used a simple and efficient method to obtain scale-aware distance. Now, we discuss the camera geometry and propose a set of losses during the process. Figure 4 shows that the whole methods we proposed in this paper.

#### 3.2.1. Pinhole Model

**Figure 5:**
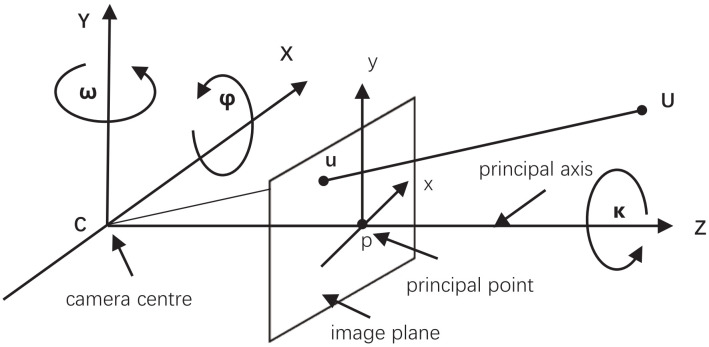
Geometric schematic of pine-hole model.

The pinhole model is a widely used model, and Figure 5 shows a geometric schematic of the pine-hole model.

#### 3.2.2. Camera Calibration

The camera pin-hole model is shown in Figure 5. To calculate the distance, we need to calibrate the camera to access the matrix of intrinsic parameters. The interrelationship between the 3D geometric position of a point on the surface of a spatial object and its corresponding point in the image is determined by the geometric model of the camera imaging, and the parameters of the geometric model are the parameters of the camera. We can use the parameter for 3D scene reconstruction, distance estimation, and other applications to realize the conversion from 2D to 3D through camera calibration.

(3) $$K=\left[\begin{array}{ccc} f_{x} & s & x_{0}\\ 0 & f_{y} & y_{0}\\ 0 & 0 & 1 \end{array}\right]$$

Here, $f_{x},f_{y}$ represent the focal lengths of the camera on the *x* and *y* axes, respectively; $x_{0}$ and $y_{0}$ are the coordinates of the main point of the image; *s* is known as skew and represents the angle of inclination of the pixel. Usually, we can use 0 to replace *s*. In the KITTI dataset, we use the official parameters. In our self-made datasets CCP (car–cyclist–pedestrian), we use the HARRIS algorithm to extract sub-pixel coordinates and use Zhang’s method to calibrate the camera. The HARRIS algorithm first implements a range of shifts for the image processing sub-window centered through a point. Then, it is expanded by first-order Taylor to obtain the value of the grayscale change of the image point before and after it has been moved. If this value can match a certain threshold size, this point is a corner point. Further, suppose the number of corner points of the image can be predetermined at calibration. An appropriate number of corner points is equal to the number of corner points to be acquired. In addition, for some larger points, which may be concentrated in some areas, the corner points will be particularly compact, but the number of corner points in a region will not be large. Therefore, it is necessary to define the maximum number of corner points in a specific area, and in this way, it is possible to effectively prevent the existence of low threshold corner points in some other areas.

When using Zhang’s calibration method, after obtaining an image of the calibration board, the pixel coordinates of each corner point (u,v) can be obtained using the corresponding image detection algorithm. The checkerboard grid of the calibration board represents the world coordinate system and the physical coordinates of any point on it *W* = 0. Since the coordinate system of the calibration board is defined artificially in advance, and the size of each grid on the calibration board is known, we can calculate the physical coordinates of each corner point in the world coordinate system. We use the above information to calibrate the camera and obtain the camera’s internal and external reference matrix.

The pose of an object, relative to the camera coordinate system, could be described in terms of the rotation matrix *R* and the translation vector *T*. Rotation around *x*, *y*, and *z* axes can be represented by rotation matrices $R_{x}$, $R_{y}$, and $R_{z}$, respectively:

(4) $$R=R_{x}R_{y}R_{z}=\left[\begin{array}{ccc} 1 & 0 & 0\\ 0 & cos_{\varphi } & -sin_{\varphi }\\ 0 & sin_{\varphi } & cos_{\varphi } \end{array}\right]\left[\begin{array}{ccc} cos_{\omega } & 0 & -sin_{\omega }\\ 0 & 1 & 0\\ sin_{\omega } & 0 & cos_{\omega } \end{array}\right]\left[\begin{array}{ccc} cos_{\kappa } & -sin_{\kappa } & 0\\ sin_{\kappa } & cos_{\kappa } & 0\\ 0 & 0 & 1 \end{array}\right]$$

Here, φ, ω, and κ are the rotation angles around the *x*, *y*, and *z* axes, respectively. Finally, the rotation matrix *R* can be composed by the multiplication of the three rotation matrices. Figure 5 shows the three variables.

#### 3.2.3. Transformation between Camera Coordinates and Image Coordinates

To calculate the distance, the transformation between camera coordinates and image coordinates is required.

(5) $$x=Kx_{c}$$

(6) $$X_{c}=\left[\begin{array}{c} \begin{array}{cc} R & T \end{array} \end{array}\right]Xw$$

As shown in Equation (5), *x* is the coordinates of the actual measurement, $x_{c}$ represent the camera coordinates, *K* is as Equation (3), and Equation (6) explains the world coordinate and the transformation of camera coordinates. $X_{c}={\left(x_{c},y_{c},z_{c}\right)}^{T}$ is the camera coordinate, and we first use Equation (5) to obtain image coordinates by projection function $X_{c}\to \Pi \left(X_{c}\right)=x$, then we use the unprojection function from the image coordinate to the camera coordinate, $\left(x,\hat{D}\right)\to \Pi^{-1}\left(x,\hat{D}\right)=X_{c}$ of an image pixel $x=(u_{0},v_{0})$, and its distance estimate $\hat{D}$ to the 3D point $X_{c}={\left(x_{c},y_{c},z_{c}\right)}^{T}$ is obtained through the following steps. Letting ${\left(x_{i},y_{i}\right)}^{T}={\left(\left(u_{0}-x_{0}\right)/k_{x},\left(v_{0}-y_{0}\right)/k_{y}\right)}^{T}$, $\left(k_{x},k_{y}\right)$ is the aspect ratio and $\left(x_{0},y_{0}\right)$ is the principal point, the distance estimate $\hat{D}$ from the network represents the Euclidean distance $∥X_{c}∥=\sqrt{x_{c}^{2}+y_{c}^{2}+z_{c}^{2}}$.

#### 3.2.4. Edge Smooth Loss and Ego Mask

As depicted in [51,52], the inverse depth map is given a regularization term because depth discontinuities often occur at image gradients. We weight this cost using the image gradients $\partial_{u_{0}}$$\partial_{v_{0}}$, and $I_{t}$ represents the image at time *t*.

(7) $$L_{s}\left({\hat{D}}_{t}\right)=\left|\partial_{u_{0}}{\hat{D}}_{t}^{*}\right|e^{-\left|\partial_{u_{0}}I_{t}\right|}+\left|\partial_{v_{0}}{\hat{D}}_{t}^{*}\right|e^{-\left|\partial_{v_{0}}I_{t}\right|}$$

${\hat{D}}_{t}^{*}={\hat{D}}_{t}/\bar{D_{t}}$, which means the inverse depth to discourage shrinking of the estimated depth. As for the ego mask, we need to remove the object which has the same velocity as the camera, such as the adjacent frames in the sequence or a low-texture region. We apply a binary per-pixel mask to the loss that selectively weights the pixels and is automatically computed on the forward pass of the network, following [30]:

(8) $$\phi =[minpe\left(I_{t},I_{t^{\prime }\to t}\right)<pe\left(I_{t},{\hat{I}}_{t^{\prime }}\right)]$$

where [ ] is the Iverson bracket. ϕ prevents the loss of contamination of pixels that remain stationary in the image.

#### 3.2.5. Resolving Scale Factor at Training Time

To resolve the simple pinhole model, our network’s output σ needs to be converted to depth with *D* = 1/(xσ+y), where *x* and *y* are chosen to constrain *D* between 0.1 and 100 units. In Section 3.2, we describe the limitations of the SFM. The monocular depth and ego-motion predicter produce a scale-ambiguous value that cannot estimate distance. Therefore, the crucial question is the pose of the camera. For training time, we use three consecutive images as a set of inputs. In the output stage, we normalize the displacement of target frame $I_{t}$, for which, using the vehicle’s velocity, the result $Po_{t\to t^{\prime }}$ is scaled by Δv.

(9) $$Po_{t\to t^{\prime }}=\frac{Po_{t\to t^{\prime }}}{∥Po_{t\to t^{\prime }}∥}\Delta v$$

#### 3.2.6. Photometric Loss

As shown in Section 3.2.3, we need to minimize the image reconstruction error from $I_{t}$ and $I_{t}^{\prime }$, distance estimate ${\hat{D}}_{t}$ at time *t*, and the pose for $I_{t}$, concerning the first image $I_{t}^{\prime }$’s pose, written as $Po_{t\to t^{\prime }}$. The point cloud $P_{t}$ can be obtained by using the distance estimate ${\hat{D}}_{t}$ of the network in the following way:

(10) $$P_{t}=\Pi^{-1}\left(p_{t},{\hat{D}}_{t}\right)$$

$\Pi^{-1}$ represents the unprojection from image to camera coordinates, and $p_{t}$ is the pixel set of $I_{t}$. The pose network outputs the $I_{t}$’s pose as function ${\hat{P}}_{t^{\prime }}$ = $Po_{t\to t^{\prime }}P_{t}$. At time *t*, we project ${\hat{P}}_{t^{\prime }}$ to the camera using the projection function Π, then, establish a mapping between the two coordinates $p_{t}={\left(u_{0},v_{0}\right)}^{T}$ and $p_{t^{\prime }}={\left(\hat{u_{0}},\hat{v_{0}}\right)}^{T}$ at time $t^{\prime }$ through the transformation and projection with shi(10). Once we obtain the mapping relation, then with the internal camera matrix, the target frame $I_{t}$ can be reconstructed by the given source frame $I_{t^{\prime }}$. The symbol represents the sampling operator, and we follow [53] to use bilinear sampling to sample the source images.

(11) $$I_{t^{\prime }\to t}=I_{t^{\prime }}\langle projection(D_{t},T_{t\to t^{\prime }},K)\rangle$$

Following previous works [51,54], we use the L1 pixel-wise loss term combined with structural similarity (SSIM) [55] to calculate the loss between the reconstruction from source image $I_{t}$ to target image ${\hat{I}}_{t^{\prime }\to t}$, where λ = 0.85 is a weighting factor. The reconstruction loss $L_{re}$ in Equation (13) is calculated through all source images.

(12) $$\tilde{L_{re}}\left(I_{t},{\hat{I}}_{t^{\prime }\to t}\right)=\lambda \frac{1-SSIM(I_{t},{\hat{I}}_{t^{\prime }\to t})}{2}+\left(1-\lambda \right)∥I_{t}-{\hat{I}}_{t^{\prime }\to t}∥$$

(13) $$L_{re}=\underset{t^{\prime }\in \left(t+1,t-1\right)}{min}{\tilde{L}}_{re}\left(I_{t},{\hat{I}}_{t^{\prime }\to t}\right)$$

Recently, the works [56,57,58] proposed new reconstruction loss functions. Hence, we combined these works and presented a robust loss function that considers multi-loss functions. We use it to replace L1 loss function in Equation (12). This dynamic loss function with parameters α and β can be transformed during deep learning, e.g., between L1 and L2, to obtain better training results. The general loss function introduces the per-pixel regression $L_{p}$; as shown in Equation (14), it is based on L1 loss, while Equation (15) is the robust loss function.

(14) $$L\left(p\right)=L\left(I_{t},{\hat{I}}_{t^{\prime }\to t}\right)$$

(15) $$L_{robu}\left(p\right)=\frac{\left|\alpha -2\right|}{\alpha }\left({\left(\frac{{\left(p/\beta \right)}^{2}}{\left|\alpha -2\right|}+1\right)}^{\alpha /2}-1\right)$$

#### 3.2.7. Attention Module in Distance Decoder

**Figure 6:**
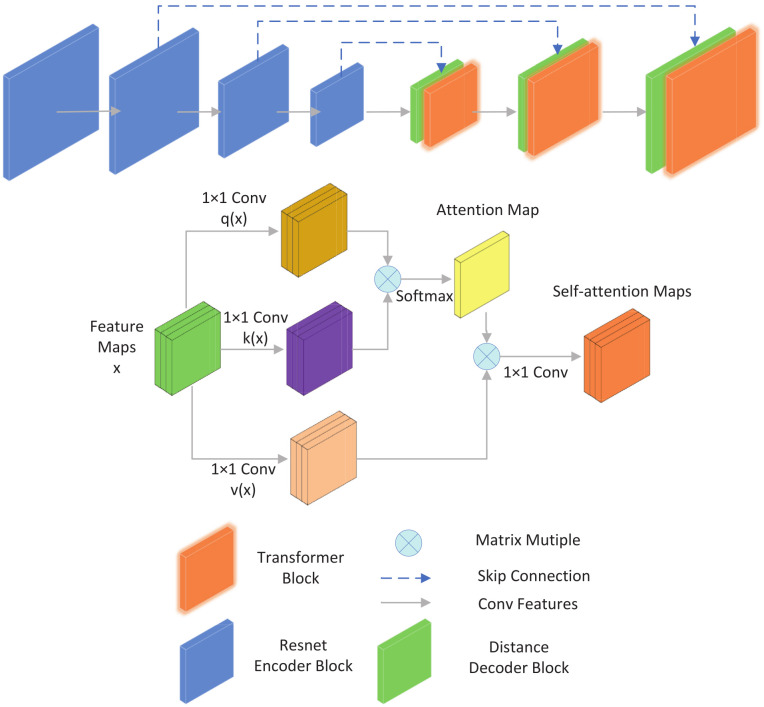
This is our depth network architecture; we use a self-attention decoder to obtain better depth maps.

In previous work [17,30,51,58], the decoded features are upsampled by nearest-neighbor interpolation or transposed convolution, which can be learned. The main drawback of this process is that since the interpolation combines the distance values of the background and foreground, it can lead to large errors in the borders of the objects in the upsampled distance map. While the attention mechanism allows the network to focus more on a certain aspect, and following [59,60], as Figure 6 shows, we tried to incorporate different attention modules in the deep graph decoder section, channel attention, spatial attention (CBAM), and self-attention. On the output feature map, local pixel regions are extracted for any pixel point $p_{ij}$ centered on a spatial range *k*, and for each region xy, the following formula is used:

(16) $$X_{ij}=\sum softmax_{xy}\left(q_{ij}^{⊤}k_{xy}\right)v_{xy}$$

As depicted in previous work [43], $q_{ij}=W_{Q}p_{ij}$ are queries, $k_{xy}=W_{K}p_{xy}$ are keys, and $v_{xy}=W_{V}p_{xy}$ are values. The matrix *W* represents different transformations, the parameters of three *W* are also different, and they can be changed through training. The softmax operation can calculate logits of local ij. As for [60], we also try to add a module on the output map. The channel and spatial attention can be better able to process marginal information. We also use pixel point $p_{ij}$ in the local region xy to test the effects. Finally, we compare the two methods and use the self-attention module.

$$\begin{array}{c} M_{ch}\left(xy\right)=\sigma \left(MLP\left(AvgPool\left(xy\right)\right)+MLP\left(MaxPool\left(xy\right)\right)\right) \end{array}$$

(17) $$\begin{array}{c} M_{sp}\left(xy\right)=\sigma \left(f^{7*7}\left(\left[AvgPool\left(xy\right);MaxPool\left(xy\right)\right]\right)\right) \end{array}$$

*MLP* represents two-weighting matrix, then a Relu activate function is needed. AvgPool and MaxPool can make every $p_{ij}$ return a feedback.

#### 3.2.8. Multi-Scale Resolution Estimation Map

Most existing models use multi-scale depth prediction to address the local gradient problem of linear samplers and the local minimal that they fall into during training, with the losses at all scales constituting the total loss of training. Inspired by previous work [61], at low resolution, distant low-texture regions in an image are displayed indistinctly, producing infinity-like distances and loss of detail. In contrast, at high resolution, the image begins to lose overall structure and produce low-frequency artifacts. Inspired by previous work [58], we output three resolutions of images, each using an attention mechanism (depicted in Section 3.2.7) and fusing them with the high-resolution image. Finally, a depth estimation image is acquired, then the object bounding box is combined, and a distance is output.

#### 3.2.9. Final Loss

As described in Section 3.2.4 and Section 3.2.5, we combine two loss functions and average over each pixel, scale, and batch through training time, $L_{tol}=\alpha L_{s}+\beta L_{robu}$.

## 4. Experiments

In this section, we train our models on the training datasets and test them on validation datasets. Moreover, we evaluate our proposed models with a comparison to alternative approaches.

### 4.1. Evaluation Metrics

Our goal is to predict a bounding box of objects and a distance as close to the ground truth as possible. Therefore, we use “precision” and “recall” to evaluate the object detection accuracy. We use four metrics to evaluate depth prediction: absolute relative difference (AbsRel), squared relative difference (SquaRel), root of mean squared errors (RMSE), and root of mean squared errors, computed from the log of the predicted distance and the log ground truth distance ($RMSE_{log}$). Let $d_{i}^{gt}$ and $d_{i}$ denote the ground truth distance and the predicted distance. We can compute the errors with the five following equations. Threshold represents the size of overlap between the prediction box and the ground truth box, and this value higher is better. The following four values represent the four ways of calculating error, and for these values, lower is better.

(18) $$\begin{array}{c} Threshold:\%\;of\;d_{i}\;s.t.\;max\left(d_{i}/d_{i}^{gt},d_{i}^{gt}/d_{i}\right)=\delta <threshold \end{array}$$

(19) $$\begin{array}{c} Abs\;Relative\;difference\left(Abs\;Rel\right):\frac{1}{N}\sum_{d\in N}\left|d-d^{gt}\right|/d^{gt} \end{array}$$

(20) $$\begin{array}{c} Squared\;Relative\;difference\left(Squa\;Rel\right):\frac{1}{N}\sum_{d\in N}||d-d^{gt}{\left|\right|}^{2}/d^{gt} \end{array}$$

(21) $$\begin{array}{c} RMSE\left(linear\right):\sqrt{\frac{1}{N}\sum_{d\in N}\left|\right|d_{i}-d_{i}^{gt}{\left|\right|}^{2}} \end{array}$$

(22) $$\begin{array}{c} RMSE\left(log\right):\sqrt{\frac{1}{N}\sum_{d\in N}||logd_{i}-logd_{i}^{gt}{\left|\right|}^{2}} \end{array}$$

### 4.2. Implementation

We first use a COCO pretrained YOLOv5 checkpoint to train our LF-YOLO. For the KITTI dataset, we set initial learning rate at 0.01, at 300 iterations, with learning rate drop by 1/10 at 200 iterations. Batch size is set to 16. The training strategy also uses a random gradient descent algorithm with a momentum term of 0.93. For the CCP dataset, we use the same parameters except the initial learning rate to train the model. We set learning rate at 0.001. In order to test the performance of our network, we use the same strategy as LF-YOLO to train YOLOv5s on the KITTI dataset, then we test the two networks in the test set.

Secondly, we train the depth network using Adam [62] with $\beta_{1}$ = 0.9, $\beta_{2}$ = 0.999, epoch is set to 30, batch is set to 16, the learning rate is 0.0001 for the first 20 epochs, then drop to 0.00001 for the last 10 epochs. The output σ of the distance decoder is converted to distance D=1/(xσ+y), where *x* and *y* are chosen to constrain *D* between 0.1 and 100 units. The input image of KITTI data is resized to 640 × 192. The smooth weight term α and photometric robust weight term β were set to 0.001. We applied channel and spatial module in the depth decoder. Compared with the self-attention module, the latter is better. We use ResNet50 as the backbone of the depth network.

The two experiments were performed on an Ubuntu 18.04 machine with a single 16 GB NVIDIA Titan-X GPU, using Pytorch 1.7.0.

### 4.3. Camera Calibration Result

**Figure 7:**
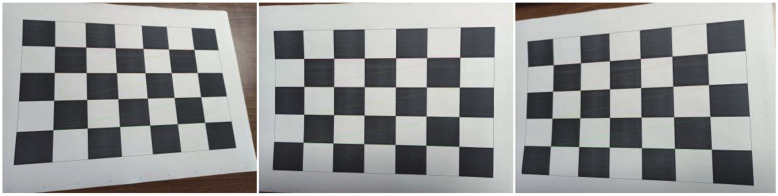
Result of camera calibration.

For the KITTI dataset, we use the official camera parameters. For the CCP dataset, we use Zhang’s method to calibrate a monocular camera. Only through the camera calibration can we obtain the camera parameters and then estimate the distance. Our calibration plate was 5 × 7 and multiple photos were taken at different angles with the camera. The internal parameters and aberration coefficients of the camera calibration were averaged over several calibrations, and the results were substituted into our network for distance estimation by the monocular camera. *K* represents the matrix of intrinsic parameters, and *d*1 represents the distortion factor. The result is shown in Figure 7.

$$K=\left[\begin{array}{ccc} 3834 & 0 & 2196\\ 0 & 3517 & 1736\\ 0 & 0 & 1 \end{array}\right]\;\;\;d1=\left[\begin{array}{cc} -1.750 & -0.142 \end{array}\right]$$

### 4.4. KITTI Dataset

We evaluate our two methods on the KITII dataset [63]. For object detection network LF-YOLO, we used 7481 training images and 7518 test images, comprising a total of 80,256 labeled objects, then grouped the categories in the KITTI target detection dataset into just three categories: cars, cyclists, and pedestrians, and used these three categories for both training and validation. LF-YOLO is slightly less accurate than YOLOv5s but nearly twice as fast, allowing more time for subsequent distance estimation and real-time detection. In addition, the inclusion of the SE module and the use of EIoU can improve precision and recall in all three classes. The final results are shown in Table 1 and Table 2, the symbol × indicates this module is not used while indicates this module is used. Table 3 shows the results of our method and other methods.

**Figure 8:**
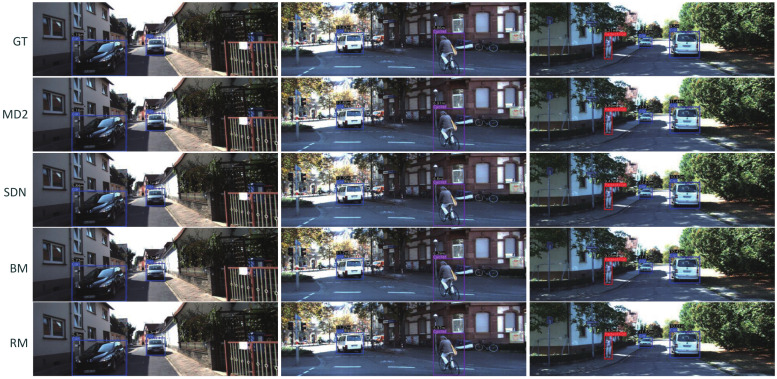
Examples of the estimated distance using our proposed base model (BM) and robust model (RM). We also provide ground truth distance (GT), Monodepth2 (MD2), and SynDistNet (SDN) for comparison.

For the distance estimate network, before training, to split raw data, we use the same method in previous work [65]. The training data contains 38,724 images, validation data contains 4597 images, and test data contains 631 images. Then, we filter static frames using the default camera matrix for all images. The focal length is averaged. Further, we add channel spatial and self-attention modules to the depth decoder. Both of them can enhance the effect. The self-attention module performs better than the CBAM module because our data are sequential, and similar objects can be better distinguished and further focused. The actual distance is acquired using the method in previous work [25]. We test our method and other classical methods, and the bold numbers in Table 3 shows that our proposed models are able to predict distances with lower absolute errors. For the classic method Monodepth2, our method is 0.014 lower than it in the absolute error of distance estimation. Moreover, while improving accuracy, we also maintain high operational efficiency. The average inference time of our model is 12.5 ms per image, which is slightly slower than Monodepth2 (14.2 ms), but twice as fast as Zhou (27.1 ms). Figure 8 shows the different results in KITTI datasets.

### 4.5. CCP Dataset

To verify the generality of our method, we performed data collection and annotation using a monocular camera with a dataset of 3482 images, manually annotated with actual distance values, each with a resolution of 1920 × 1080. The camera was calibrated using Zhang’s method in [66], including both intrinsic and extrinsic parameters. The dataset was divided into 3113 images used for training and 369 for testing. Training on this dataset was carried out in a similar way to KITTI. The initial learning rate was set to 0.001, the input size was adjusted to 640, and $L_{robu}$ loss-supervised distance regression was used. The test results are shown in Table 4 and our proposed method also achieved more accurate results (the bold numbers).

### 4.6. Ablation Studies

We conducted an ablation study to evaluate the distance estimation network. The specific results are shown in Table 5, where the accuracy was improved after replacing the traditional L1 loss with a robust loss function. The self-attention module has a more significant improvement for the network than CBAM. The former fully considers the location and content information and uses a multi-scale approach to generate the estimated distance map from the information fusion. We can see that by adding the above modules, the network’s performance can be continuously improved.

## 5. Conclusions

In this paper, we propose a deep neural network that simultaneously implements object detection and object distance estimation. We calibrate a monocular camera and use it to capture images and calculate distance. The proposed LF-YOLO is a variant based on Shufflnetv2 and YOLOv5 for distance detection. A distance estimation network is proposed, which treats the camera parameters as part of the input to the network, uses a new loss function for self-supervised training, uses a multi-scale approach for distance map fusion, and finally combines the target detection results for distance output. Experimental results on two datasets show that our proposed network can estimate object distances accurately, it outperforms existing algorithms for depth and object estimation, and it operates with high efficiency.

## Figures and Tables

**Table 1:** Detection results for different network targets.

Class	Method	EIoU	SE Layer	Precision (%)	Recall (%)
Car	YOLOv5s	× ✓	-	94.4 95.3	90.2 91.3
	LF-YOLO	× ✓ × ✓	× × ✓ ✓	88.0 89.5 90.3 92.4	84.1 84.9 87.4 88.7
Pedestrian	YOLOv5s	× ✓	-	95.0 95.6	93.5 94.0
	LF-YOLO	× ✓ × ✓	× × ✓ ✓	90.1 90.9 92.5 93.2	85.6 86.6 89.3 89.9
Cyclist	YOLOv5s	× ✓	-	90.3 91.0	81.5 83.1
	LF-YOLO	× ✓ × ✓	× × ✓ ✓	80.2 82.9 83.0 86.1	75.8 77.2 78.7 79.6

**Table 2:** Results for different network operating efficiencies.

Method	SE Layer	Infer Time per Frame (ms)	Parameters
YOLOv5s	-	94.5	7.3 M
LF-YOLO	×	47.5	2.1 M
	✓	56	3.9 M

**Table 3:** Quantitative performance comparison of our network with other self-supervised monocular methods for the KITTI dataset.

Approach	Lower Is Better			Higher Is Better		
	AbsRel	SquaRel	${\mathrm{RMSE}}_{\log}$	δ < 1.25	δ < $1.25^{2}$	δ < $1.25^{3}$
Zhou [30]	0.183	1.595	0.270	0.734	0.902	0.959
GeoNet [17]	0.149	1.060	0.226	0.796	0.935	0.975
Struct2depth [64]	0.141	1.026	0.215	0.816	0.945	0.979
PackNet-SfM [19]	0.120	0.892	0.196	0.864	0.954	0.980
Monodepth2 [58]	0.115	0.903	0.193	0.877	0.959	0.981
FisheyeNet [50]	0.117	0.867	0.190	0.869	0.960	0.982
SynDistNet [57]	0.109	0.718	0.180	0.896	0.973	0.986
Shu [18]	0.104	0.729	0.179	0.893	0.965	0.984
Ours	**0.101**	**0.715**	**0.178**	**0.899**	**0.981**	**0.990**

**Table 4:** Evaluation results on CCP dataset.

Method	Lower Is Better			Higher Is Better		
	AbsRel	SquaRel	${\mathrm{RMSE}}_{\log}$	δ < 1.25	δ < $1.25^{2}$	δ < $1.25^{3}$
Baseline	0.198	1.034	0.241	0.841	0.886	0.910
Baseline + Att	0.163	0.894	0.192	0.851	0.897	0.924
Baseline + $L_{robu}$	0.154	0.887	0.190	0.849	0.891	0.923
Baseline + Att + $L_{robu}$	**0.121**	**0.723**	**0.181**	**0.872**	**0.915**	**0.941**

**Table 5:** Ablation study on different variants of our network using the KITTI dataset. $L_{robu}$, SA, CBAM, and MRE represent robust loss function, self-attention module, channel and spatial attention module, and multi-scale resolution estimation.

Method	$L_{\mathrm{robu}}$	SA	CBAM	MRE	AbsRel	SquaRel	${\mathrm{RMSE}}_{\log}$	δ < 1.25
Ours	×	×	×	×	0.205	1.617	0.282	0.812
Ours	✓	×	×	×	0.154	1.129	0.216	0.848
Ours	✓	✓	×	×	0.121	0.812	0.191	0.874
Ours	✓	×	✓	×	0.130	0.856	0.208	0.859
Ours	✓	✓	×	✓	0.101	0.715	0.178	0.899

## Data Availability

The data present in this study are openly available at https://ieeexplore.ieee.org/document/6248074/, accessed on 26 July 2012.
